# Efficacy and Mechanism of Thymol/KGM/LG Edible Coating Solution on Inhibition of *Mucor circinelloides* Isolated From Okra

**DOI:** 10.3389/fmicb.2022.880376

**Published:** 2022-05-16

**Authors:** Qinqiu Zhang, Wen Qin, Xinjie Hu, Jing Yan, Yaowen Liu, Zhuwei Wang, Lang Liu, Jie Ding, Peng Huang, Jiya Wu

**Affiliations:** ^1^Sichuan Key Laboratory of Fruit and Vegetable Postharvest Physiology, College of Food Science, Sichuan Agricultural University, Ya’an, China; ^2^College of Food Science and Technology, Sichuan Tourism University, Chengdu, China; ^3^Department of Quality Management and Inspection and Detection, Yibin University, Yibin, China

**Keywords:** okra, *Mucor circinelloides*, pathogens, Thymol/KGM/LG edible coating solution, antifungal mechanism

## Abstract

With the increasing demand and quality requirement for the natural nutritious food in modern society, okra has attracted much attention because of its high nutritional value and remarkable functionality. However, the occurrence of postharvest diseases of fresh okra severely limited the application and the value of okra. Therefore, in this study, the dominant pathogens causing postharvest diseases such as soft rot were isolated from naturally decaying okra. It was identified as *Mucor circinelloides* by its morphological characteristics and standard internal transcribed spacer ribosomal DNA sequence. Furthermore, the biological characteristics of *M. circinelloides* were studied, and the inhibitory effect of thymol/KGM/LG (TKL) edible coating solution on *M. circinelloides* and its possible mechanism was discussed. In addition, TKL edible coating solution had a dose-dependent inhibitory effect on *M. circinelloides*, with a 50% inhibitory concentration (EC50) of 113.55 mg/L. The TKL edible coating solution at 960 mg/L of thymol completely inhibited mycelial growth and spore germination of *M. circinelloides*. The results showed that the best carbon source of *M. circinelloides* was maltose, the best nitrogen source was beef extract and potassium nitrate, the best pH was 6, the best temperature was 28°C, the best NaCl concentration was 0.5%, and the light was conducive to the growth of *M. circinelloides*. It was also observed by scanning electron microscope (SEM) that TKL was more likely to destroy the cell wall integrity of *M. circinelloides*, inhibit spore morphology and change mycelium structure. Meanwhile, the activity of chitinase (CHI), an enzyme related to cell wall synthesis of *M. circinelloides*, was significantly decreased after being treated by TKL with thymol at 100 mg/L (TKL100). The content of Malondialdehyde (MDA) in *M. circinelloides* decreased significantly from 12 h to 48 h, which may cause oxidative damage to the cell membrane. The activity polygalacturonase (PG), pectin methylgalacturonase (PMG), and cellulase (Cx) of *M. circinelloides* decreased significantly. Therefore, the results showed that TKL had a good bacteriostatic effect on okra soft rot pathogen, and the main bacteriostatic mechanism might be the damage of cell membrane, degradation of the cell wall, inhibition of metabolic activities, and reduction of metabolites, which is helpful to further understand the inhibitory effect of TKL on okra soft rot pathogen and its mechanism.

## Introduction

Okra is a valuable herb of the genus okra in the mallow family, which is widely grown in tropical and subtropical regions (Dantas et al., 2021). Okra has many medicinal properties, such as antifatigue, enhancing immunity, protecting the liver and kidney, reducing lung injury, antihypertension, lowering blood lipid, lowering blood pressure, anticancer, and so on (Elkhalifa et al., 2021a). Young okra pods are commonly eaten as vegetables and raw materials for polysaccharide extraction, okra seeds are utilized for oil and protein extraction, and aging fruit pods are served as animal feed (Fekadu Gemede, 2015). In particular, young okra pods are delicious, tender and lubricating, low in energy, free of oil, and contains high okra polysaccharide, rich in carotene, vitamin C, a variety of B vitamins and mineral elements such as Fe, Mn, and Zn, as well as rich phenolic compounds, such as oligocatechin and hydroxybenzoic acid derivatives (Elkhalifa et al., 2021b; Uddin Zim et al., 2021). However, due to the various microbial factors and plant characteristics, okra is particularly susceptible to spoilage during postharvest storage (Tsado, 2015). For example, fungal rot caused by *Fusarium Solani* (Kwon and Jee, 2005; Kapadiya et al., 2014) and brown rot caused by *Anthrax pathogen genus* (Farrag, 2011). Studies have found that the tissue of okra is easy to soften after the harvest, and the juice oozes out and becomes mildew and rot, temporarily called soft rot, which causes great economic loss of okra (Tohyama et al., 1995). However, the limited studies that reported the causes of postharvest soft rot of okra, and the related pathogenic mechanism and control.

In recent decades, the plant extracts have been widely used in the prevention of fruit and vegetable diseases and the development of green fruit and vegetable preservatives, they are aromatic and volatile components extracted from specific plant parts, most of which have antifungal effects (Kaur et al., 2021). The common monomer plants such as cinnamaldehyde (Li et al., 2019; Han et al., 2021), carvacrol (Chen et al., 2021), menthol (Yang et al., 2020), thymol, and vanillin (Florencia Bambace et al., 2019) have been applied to the control of fruit and vegetable diseases and preservations and achieved good results (Hyldgaard et al., 2012; Nazzaro et al., 2013). It is worth mentioning that thymol has been applied in various forms to the prevention and control of diseases and storage of fruits and vegetables in recent years, especially for the prevention and control of postharvest soft rot and brown spot (Sun et al., 2021; Selvam et al., 2022). In addition, thymol has been approved by the United States Food and Drug Administration (FDA) for use in foods as a food additive, and it is also the European Commission has registered oils, is a recognized safety material, non-toxic side effects on the mammalian physiological system, can be used as a flavoring agent and preservative in food, are widely used in pharmaceutical, food and beverage production (Nagoor Meeran et al., 2017). Unfortunately, thymol is unstable, water-insoluble and it has unsatisfactory properties. Encapsulation of thymol into microcapsules is one of the effective alternatives to overcome these shortcomings (Serna-Escolano et al., 2020).

This study first isolated the main pathogenic bacteria in postharvest soft rot in okra, and with thymol, emulsifier, β-cyclodextrin, and polyethylene glycol (PEG), namely, PEG6000, as the main raw materials mixed in proportion, thymol microcapsule preparation was done by spray drying powder, which was supplemented with konjac glucomannan (KGM) and low acyl knot gels (LG) that were prepared from the matrix liquid, thymol/KGM/LG (TKL) edible coating solution was formed. At present, the laboratory where we worked on this study has applied for the relevant invention patent, “A kind of thymol compound biological coating preservative and its preparation method and application” (CN202010919222.9). And using TKL as an antibacterial substance, the inhibitory effect on the main pathogenic bacteria of okra soft rot was further explored., and preliminarily explore the related antifungal mechanism. It provides a new idea and method for disease control and postharvest storage of okra and is expected to reduce the loss caused by postharvest fresh okra decay and improve economic benefits.

## Materials and Methods

### Materials and Chemicals

Fresh okra was grown in Chengdu country, Sichuan Province, China, and it was harvested. The spoiled okra was provided to isolate the pathogen in the Sichuan Agricultural University of China. Potato dextrose agar (PDA) (Beijing AoBoXing bio-tech Co. Ltd., Beijing, China) was used as the culture media. Thymol (98.00% purity) was purchased from Shanghai Ron Reagent or Shanghai Yien Chemical Technology. All other chemicals and reagents were of analytical grade and they were purchased from Chengdu Cologne Chemical Reagent Company (Chengdu, China).

Thymol microcapsule powder was equipped with 1-L thymol microcapsule emulsion, a total of 5.28-g thymol; 10-g emulsifier; β-cyclodextrin, 123.15 g; PEG6000, 61.57 g; and 1-L ultra-pure water. The β-cyclodextrin was dissolved in 500-ml hot water over 75°C and then stirred in a magnetic stirrer (80°C) at 600 rpm/min. Also, PEG was dissolved by adding 300-ml hot water over 75°C in a high-speed stirrer at 1000 rpm/min and then placed in an ultrasonic cleaning tank for 15 min to exhaust. After the emulsifier was dissolved in 200-ml hot water over 75°C, the temperature was kept warm in a 60°C water bath. The exhaust PEG solution was added into the β-cyclodextrin solution, and the magnetic stirrer temperature was controlled over 75°C, 600 rpm/min, and stirred for 30 min. Also, adjusted the temperature to 60°C. Added emulsifier solution holding at about 60°C until uniform solution was arrived at. Then cool downed to about 40°C, and added core material (two drops per second). After stirring for 3 h (40°C, 600 rpm/min), microcapsule powder was obtained after spray drying. The effective content of muscimol in the prepared microcapsules was 26.1 mg/g.

The preparation of membrane matrix solution was as follows: Boiled 500-ml ultrapure water along with 0.125-g KGM and 0.25-g LG. High-speed agitator (1,200–1,600 rpm/min), stirred for 30 min; added 0.2 g calcium stearate lactate and continued to stir for 5–10 min. Covered with plastic wrap and brought to a boil. The uniform membrane solution was obtained by ultrasound for 30 min (60°C, 80 W). The TKL mother liquor was equipped with 10-ml uniform membrane matrix solution along with 1.88679-g of microcapsule powder; after mixing, a centrifugation for 30 min and vortex for 5–10 min to obtain uniform mother liquor (concentration of 5 mg/ml); the TKL with different concentration gradients was diluted with membrane matrix.

### Isolation and Identification of Pathogens

According to the plant tissues separation method (Jogee et al., 2017), the spoiled okra about 6 mm^2^ was excised from diseased berries with a sterile blade at the junction of the diseased and healthy sarcocarp. Each piece of sarcocarp was washed in sterile double-distilled water (SDW) and it was disinfested in 75% ethanol for 30 s and 1% sodium hypochlorite for 30 s, and then it was rinsed three times in SDW. After an incubation at 28°C for 48 h, the micromorphological characteristics of the colonies were observed, namely, color, growth diameter, texture, mycelial size, and conidial shape of the colonies. After the purification, the fungi were cultured on PDA and stored at 4°C for subsequent identification.

The mycelia of the tested strains were scraped and cultured at 28°C for 1–2 days on a PDA plate, and DNA was extracted using TSINGKE Plant DNA Extraction Kit (universal type). The extracted DNA samples were diluted and used as polymerase chain reaction (PCR) template and 1 × TSE101 gold mix for the amplification. The components of the amplification system were as follows: 1 × TSE101 gold Mix 45 μL, ITS1 (10P) 2 μL, ITS4 (10P) 2 μL, DNA template 1 μL. The reaction conditions were as follows: 98°C 2 min, 98°C 10 s, 56°C 10 s, 72°C 10 s/kB, 35 cycles; 72°C for 5 min, stored at 4°C. The amplified PCR products were subjected to agarose gel electrophoresis (2 μL sample + 6 μL bromophenol blue) at 300-V voltage for 12 min to obtain the identification gel pattern. The PCR products will be prepared for generation sequencing (sequencing primer is ITS). The work was commissioned by the Chengdu Branch of Beijing Qingqing Biotechnology Co., Ltd.

Contig express was used to join the sequencing results, and the inaccurate parts at both ends were removed. The spliced sequences were compared in the National Center for Biotechnology Information (NCBI) database (bla. NCBI. nLM. Nih.gov), and the species with the highest homology were selected to construct the related phylogenetic tree using MEGA 5.02 software.

### Pathogenicity Determination

A 6-mm *M. circinelloides* was inoculated into healthy okra by puncture inoculation. A sterile PDA (6 mm) was used as blank groups and inoculated into okra by puncture inoculation, which was cultured in a constant temperature incubator at 28°C. The postharvest symptom of *M. circinelloides* on okra was observed, which was confirmed as pathogenic bacteria compared with blank.

### Observation of Biological Characteristics of Pathogenic Bacteria

The isolated and identified pathogenic bacteria were inoculated on a PDA plate for a dark culture at 28°C for 3–7 days, and bacteria cakes with diameters of 6 mm were taken to a PDA medium. The different carbon and nitrogen sources, temperature, pH value, NaCl concentration, and light conditions were set up in the experiment. After 3–5 days, the colony diameter was measured by the cross-crossing method. The effects of carbon and nitrogen sources on the colony growth and sporulation were studied. Eight carbon sources were tested using the Sabouraud Dextrose Agar medium as the basal medium as follows: Glucose, maltose, agarose, lactose, starch, fructose, sucrose, and soluble sugar. Five kinds of medium were prepared by replacing the carbon sources with equal amounts of glucose. Six nitrogen sources were selected as follows: Urea, ammonium nitrate, peptone, potassium nitrate, ammonium sulfate, and beef extract, according to the amount of peptone replacement. The cake was transferred to the plate center with different carbon and nitrogen sources, and a dark culture was conducted at 28°C. The influence of culture temperature on colony growth and sporulation was studied. The cakes were transferred to the center of a new PDA plate and placed in the dark culture at 15, 24, 28, and 35°C, respectively. The influence of pH on the colony growth and the spore production was studied. The pH value of the PDA medium was adjusted with 0.1 mol/L hydrochloric acid and 0.1 mol/L sodium hydroxide solution. After sterilization, the prepared cake was transferred to the center of the PDA plate with different pH values (5.0, 6.0, 7.0, 8.0, and 9.0). A dark culture at 28°C was conducted. The effects of light conditions on the colony growth and the sporulation. The light conditions were set as a dark culture; a light culture and an alternating light and dark cultures (light and dark for 12 h each); PDA culture medium; and the culture temperature were maintained at 28°C. The effects of different NaCl (%) on colony growth and spore production were studied. The colony diameter was determined by using PDA medium containing 0, 0.5, 1.0, 1.5, 2.0, 2.5, and 3.0% NaCl at 28°C for 72 h.

### The *in vitro* of Antifungal Activity of Thymol/KGM/LG on Mycelial Growth and Spore Germination

The effects of the different concentrations of TKL on the growth of pathogenic bacteria were determined by the growth rate method. Also, 0, 400, 800, 1,600, and 2,000 μl of 5-mg/ml TKL mother solution (the solvent was polysaccharide membrane matrix, and the control group was PDA and blank PDA with 1-ml membrane matrix) were added into 100-ml PDA medium, respectively. The final mass concentration of thymol was 0, 20, 40, 60, 80, and 100 mg/L, respectively, and then mixed into sterile Petri dishes for later use. A sterile hole punch with a diameter of 6 mm was used to make holes of 6 mm diameter in different concentrations of TKL Petri dishes and control Petri dishes, respectively. Then a sterile hole punch with a diameter of 6 mm was used to cut bacterial cakes from pathogen dishes cultured for 2 days, and the cakes were embedded in the holes of TKL Petri dishes with different concentrations of TKL and control Petri dishes. The culture dish was placed in a constant temperature incubator at 28°C for 3–5 days. The colony diameter was measured by the cross-crossing method, and the inhibition rate of TKL coating on mycelial radial growth was calculated according to the formula below.


$$\mathrm{Growth}\,\mathrm{Inhibition}\,\mathrm{rate}\%=\frac{\begin{array}{c} \mathrm{Colony}\,\mathrm{diameter}\,\mathrm{of}\,\mathrm{control}\,\mathrm{group}\,\left(\mathrm{mm}\right)\\ -\mathrm{Colony}\,\mathrm{diameter}\,\mathrm{of}\,\mathrm{treatment}\,\mathrm{group}\,\left(\mathrm{mm}\right) \end{array}}{\begin{array}{c} \mathrm{Colony}\,\mathrm{diameter}\,\mathrm{of}\,\mathrm{control}\,\mathrm{group}\,\left(\mathrm{mm}\right) \end{array}}\times 100\%$$

Distilled water, film-forming matrix, and anhydrous ethanol were used as the negative control, and thymol ethanol solution with equal concentration was used as the positive control. A 200-μL of the prepared spore solution (10^5^ CFU/ml) was placed in a 96-well plate, and the final concentrations of thymol were 0, 40, 80, 100, 120, 240, 480, 960, 1920 mg/L, respectively. The treated 96-well plates were placed in a constant temperature and humidity incubator for 72 h at 28°C, and the OD600 value of each treatment was detected by a microplate tester. The inhibitory effect of thymol on the growth of pathogenic bacteria was measured by the value. The minimum inhibitory concentration (MIC) of thymol on the growth of pathogenic bacteria was obtained according to the inhibitory effect.

### Inhibition of Thymol/KGM/LG on the Mycelia Weight of *Mucor circinelloides*

(1) Preparation medium (PDA liquid): Peeled potatoes, diced and weighed 200 g; boiled with 1 L of ultra-pure water for 20–30 min; filtered with two layers of gauze; and the filtrate was taken. Added 20-g glucose; dissolved it in a constant volume to 1 L; packed it separately in 250-ml and 500-ml conical bottles, 100 ml each, and sterilized. When cooling to about 55°C, added 5% tartaric acid or 10% lactic acid (filtration sterilization), adjust pH to 3.5.

(2) Determination of fungal growth curve: (I) Preparation of bacterial suspension: Added 5 ml normal saline to the activated bacterial species incline. Scraped off the bacterial lichen or spores with inoculation ring to make bacterial suspension or spore suspension, dilute counting, suspension controlled at 10^5^–10^6^ CFU/ml. (II) Fungi were cultured in a 50 ml liquid medium in a 300-ml conical flask, and TKL was added to the experimental group. The concentration of thymol in the liquid medium was 100 mg/L, and 50-μL fungal suspension or spore suspension (10^6^ CFU/ml) was inoculated in the shaking table at 28–30°C for 140 rpm/min. The growth of fungi was measured by the dry weight method. The triangular flask was taken out at intervals of 12 or 24 h (48 h or according to the actual growth of bacteria), and the culture medium was pumped and filtered on quantitative filter paper. The medium was baked at 60°C until it reaches a constant weight, and the dry weight of the bacteria was weighed. The dry weight of mycelia = the weight of membrane after dry weight of membrane before filtration. (III) The growth curve according to the incubation time (h) and dry weight (g) was drawn.

### The Effects of Thymol/KGM/LG on the Structure of Pathogenic Bacteria Were Observed by Scanning Electron Microscope

Cover fragment culture of bacteria: (1) Water agar plate cooling set aside. Melted PDA solid medium to a liquid state, and kept the temperature of 50–60°C, which was poured into the plate; picked up the sterile cover glass with tweezers, gently dipped it into the melted PDA medium, and quickly pulled it out. A very thin medium film was adhered to the cover glass and solidify in a moment. (2) Placed cover slides horizontally on the surface of a flat plate containing sterile 1.5% water agar. Placed a maximum of four cover slides on a flat plate, but be careful not to allow them to overlap with each other. The agar with 1.5% solid water mainly acted as a wet chamber, which was providing humidity during the fungal growth. (3) Inoculated the fungus in the center of the cover glass with medium film with an inoculation needle (click lightly). All the above steps were carried out under aseptic conditions, covering the lid, and culture for 48 h in the incubator at 28°C 90% RH constant temperature and humidity (culture time is different for different strains, the specific end of culture was the beginning of spore production). (4) After the strains were matured, the cover glass was removed and put into a sterile empty medium. A 2% glutaraldehyde was dropped on the cover glass (light drops, and the entire colony and spores were submerged) and fixed for 2 h in a refrigerator at 4°C. (5) Removed the cover glass and wash it with sterile phosphate buffered solution (PBS) solution in a sterile Petri dish three times, 10 min each; Then soak in 1% osmium solution at 4°C for 2 h (this step could be omitted). The sample was placed in 50, 70, 95, and 100% alcohol to dehydrate and replace; the time was generally 10–15 min for each grade. Iso–amyl acetate was substituted at 4°C for 20 min. All these soaking steps were carried out in clean Petri dishes at 4°C. (6) Placed the cover glass after a treatment in a clean Petri dish (labeled Petri dish), and placed it at –80°C for rapid pre-cooling for 12 h (overnight). (7) The samples were freeze-dried in a vacuum (36–48 h) and treated with vacuum gold plating before scanning electron microscopy. After critical point drying and coating, the results were observed by scanning electron microscope and photographed (equipment model: ELECTRON microscope; United States FEIF50 energy spectrum: United States EDAX OCTANE SUPER. This step was operated by Etesting company). The method could observe the fungal spores and mycelia in the natural state.

### Effects of Thymol/KGM/LG on the Intracellular Structure and Enzyme Activity of *Mucor circinelloids*

Configure medium (PDA liquid): Configured potato glucose liquid medium according to the corresponding requirements. Cultivation of fungi and preparation of bacterial suspension. A 5-ml normal saline was added to the inclined plane of activated bacterial species, and bacterial suspension or spore suspension was prepared by scraping off the bacterial lichen or spores with inoculating ring, and the suspension was controlled at 10^5^–10^6^ CFU/ml. The fungi were cultured and filled with 50-ml liquid medium in a 300 ml Conical flask, which was divided into (i) experimental group and (ii) blank control group. The experimental group was added with 600-μl 5-mg/ml TKL coating solution, and the thymol concentration in the PDB medium was 100 mg/L and mixed evenly. Inoculated with 50-μL fungal suspension or spore suspension (10^5^–10^6^ CFU/ml) and cultured in a 28°C 140 rpm/min shaking table. The samples were taken at 24 h for the first time and at intervals of 12 h for 24, 36, 48, and 60 h. The culture medium was filtered, the thallus was cleaned, and then frozen with liquid nitrogen and stored in a refrigerator at −80°C for future use. In sample preparation, the frozen thistle was ground into fine powder under the condition of low temperature maintained by liquid nitrogen. A 1 g of thistle powder was added into 9 ml of pre-cooled PBS (0.01 M, pH 7.4) solution, centrifuged at 4°C for 30 min at 5,000*g*, and the supernatant could be detected. The enzyme activity was determined by MDA, PG, PMG, β-1.3-glucanase, chitinase (CHI), and Cx kits (these kits were purchased from Jiangxi Jingmei Biotechnology Co., Ltd, Yancheng city, Jiangsu Province, China.).

### Statistical Analysis

The results were expressed as means ± standard deviation (SD) of five independent replicates, with no significant difference between treatment and experimental variables. Statistically significant differences between the mean values were analyzed with one-way analysis of variance (ANOVA) and Duncan’s multi-range test using SPSS 25.0 (IBM, NY, United States). Differences at *p* < 0.05 were indicated significant.

## Results

### Isolation and Identification of Pathogenic Bacteria

**FIGURE 1:**
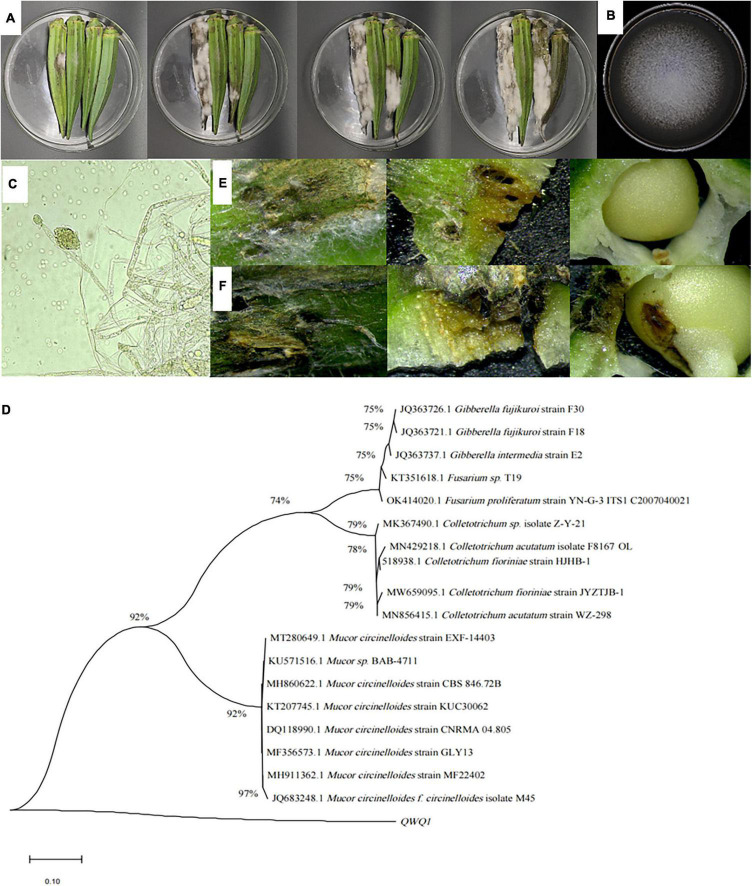
Symptoms of soft rot of okra **(A)**, and morphological **(B,C)** characteristics of the isolated pathogen, as well as its phylogenetic tree **(D)**, and observation of okra reverse connection **(E,F)**. **(A)** Symptoms on the surface of diseased okra; **(B)** colonies of pathogen cultured for 5 days at 28°C; **(C)** mycelia of pathogen and 100× magnification; **(D)** phylogenetic analysis of DNA sequences obtained from fragments of the ITS rDNA from the isolate along with the reference sequences from NCBI. The analysis was conducted using the maximum likelihood method. The scale bar represents 0.01% substitutions of nucleotide. **(E)** Okra symptoms were observed 36 h after the puncture inoculation. **(F)** Okra symptoms were observed 36 h after puncture inoculation.

The lesions of soft rot okra were disseminated from the surface to the inside and spread to the surrounding areas, showing black–brown to black, with depression and folds on the surface, brown plaque and a large number of mycelia, extravasation of juice, obvious soft tissue, and the pathogenic tissue changed from green to black–brown (Figure 1A). Then the entire tissue rotted away. After 7 days, the pathogen colonies on PDA were round and grayish–white with soft, fluffy, and grayish–white texture (Figure 1B). The aerial mycelia radiate from the center to the periphery. The mycelia were transparent and spaced, curly, and folded. Oval spores produced a pale–yellow spore pile; the spore was a single colorless, transparent, spore head into grain shape, and light yellow one (Figure 1C).

To further identify the isolated pathogen, ITS 1-5.8S-ITS region 4 was sequenced. The ITS sequence was preliminarily analyzed and compared with NCBI sequences with a similarity greater than 99% using BLAST, and two sets of exogenous sequences were introduced; MEGA 5.02 software was used to construct the phylogenetic tree and analyze the sequence using the maximum likelihood method. According to the sequence comparison of the ITS1-5.8S-ITS4 region, the isolated pathogenic bacteria had the highest similarity with *M. circinelloides* (Figure 1D).

### Pathogenicity Determination

The pathogen *M. circinelloide*, which caused okra soft rot, was verified by the method of cake puncture inoculation and pathogenicity test. The brown spots appeared on the okra surface at 36 h (Figure 1E), and mycelium began to distribute in tissues at 60 h (Figure 1F), softening, decay and depression appeared in tissues, and the surface color became dark, which were disease spots. At the same time, the mycelium penetrated the internal tissues, resulting in an internal decay.

### Biological Characteristics of *Mucor circinelloides*

**FIGURE 2:**
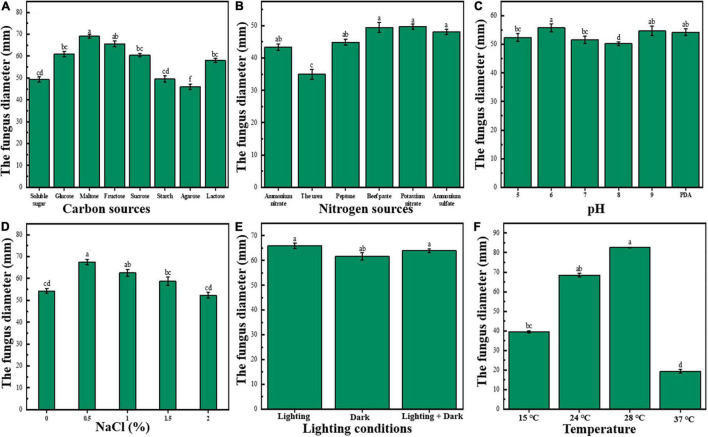
Biological characteristics of *M. circinelloides* **(A–F)**. **(A)** Effects of different carbon sources on growth of *M. circinelloides*. **(B)** Effects of different nitrogen sources on growth of *M. circinelloides*; **(C)** Effects of different pH on growth of *M. circinelloides*. **(D)** Effects of different NaCl concentrations on the growth of *M. circinelloides*. **(E)** Effects of different light conditions on growth of *M. circinelloides*. **(F)** Effects of different temperatures on the growth of *M. circinelloides*.

The effects of different carbon sources on colony growth are discussed as follows: The pathogenic bacteria could grow on all the above eight carbon sources, but there were differences in the growth of different carbon sources (Figure 2A). Also, *M. circinelloides* grew fastest when maltose was used as a carbon source; the colony diameter reached 69.07 ± 0.85 mm after incubation at 28°C for 3 days, followed by fructose (*p* < 0.05), sucrose, glucose, and lactose that had no significant difference; starch and soluble sugar also had no significant difference; and agarose grew the slowest when used as carbon source. In conclusion, there were certain differences in the absorption and utilization capacity of pathogenic bacteria to different carbon sources.

The effects of different nitrogen sources on bacterial colony growth are discussed as follows: The pathogenic bacteria could grow on the above six nitrogen sources, but there were significant differences among different nitrogen sources (Figure 2B). Among them, *M. circinelloides* had the best mycelium growth on beef extract and potassium nitrate (*p* < 0.05), followed by ammonium sulfate, peptone, ammonium nitrate, and urea. Among them, the growth rate on urea was the slowest, and the growth rate was much lower than that of other groups. Therefore, the best nitrogen sources are potassium nitrate and beef extract.

The effects of different pH on colony growth are discussed as follows: *Mucor circinelloides* could grow well in the Ph range of 5.0–9.0 (Figure 2C). When there was a pH of 6.0, the colony diameter was the largest and the mycelial growth rate was relatively the highest (*p* < 0.05), it could be seen that pH 6.0 was the optimal pH value for mycelium growth; *M. circinelloides* might be suitable to grow in an acidic environment.

The effects of different NaCl (%) on colony growth are discussed as follows: *Mucor circinelloides* could grow well in the range of 0–2.0% NaCl concentration (Figure 2D). When the concentration is 0.5%, the colony diameter reaches the maximum (*p* < 0.05), the mycelium growth rate was relatively the highest, and there was no significant difference between 2.0% and the blank group. Also, 1.0 and 1.5% could significantly promote growth. Therefore, the addition of NaCl less than 2% in the culture medium may be beneficial to the growth of *M. circinelloides*.

The effects of different light conditions on colony growth are discussed as follows: See Figure 2E. Under full light, the colony diameter was the largest, but there was no significant difference with a half-light, and there was a significant difference with full darkness (*p* < 0.05), indicating that light was beneficial to the growth of *M. circinelloides*.

The effects of different temperatures on colony growth are discussed as follows: See Figure 2F. As can be seen from the figure, *M. circinelloides* can grow in the temperature range of 15–37°C. The optimum growth temperature of *M. circinelloides* mycelia was 28°C (*p* < 0.05); too high or too low temperature might inhibit its growth and reproduction.

### Inhibitory Effect of Thymol/KGM/LG on *Mucor circinelloides in vitro*

**FIGURE 3:**
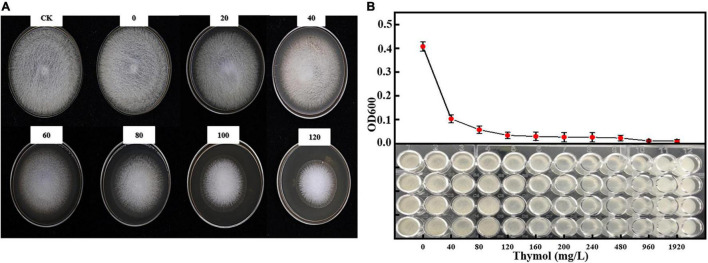
Effect of different concentrations of thymol on mycelial growth **(A)** and spore germination **(B)** of *M. circinelloides*. Values are the average of the replicates for all the analyses**. (A)** Inhibitory effect of different concentrations of TKL on *M. circinelloides*; **(B)** Inhibitory effects of different concentrations of TKL on spore germination of *M. circinelloides*.

We evaluated the antifungal activity of TKL against *M. circinelloides*, which was sensitive to thymol in TKL. Compared with the untreated group, the growth of *M. circinelloides* treated with different concentrations of TKL was inhibited, and the higher the concentration, the better was the inhibition effect. The results showed that 20–120-mg/L TKL edible coating solution had a significant effect on the growth of mycelia (*p* < 0.05; Table 1 and Figure 3A), and the median inhibitory concentration was 113.55-mg/L. According to the RESULTS of OD600 value, 40–960 mg/L TKL could significantly inhibit the conidial germination of *M. circinelloides* (Figure 3B). With the increase of concentration, the inhibition effect became better. When the concentration reached 960 mg/L, TKL could completely inhibit the conidial germination.

**TABLE 1:** Inhibitory effect of different concentrations of Thymol/KGM/LG on *Mucor circinelloides*.

Bacteriostatic agents	Thymol concentration	The fungus diameter	Inhibition rate (%)
CK	0 mg/L	77.65 ± 1.65^ab^	0.00
TKL (0)	0 mg/L	86.26 ± 0.84^a^	–12.01
TKL (20)	20 mg/L	76.21 ± 1.09^ab^	2.01
TKL (40)	40 mg/L	70.64 ± 1.70^bc^	9.79
TKL (60)	60 mg/L	64.09 ± 2.39^cd^	18.94
TKL (80)	80 mg/L	50.91 ± 2.46^de^	37.32
TKL (100)	100 mg/L	45.95 ± 0.77^ef^	44.25
TKL (120)	120 mg/L	38.80 ± 0.58^f^	54.23

*Measurement of colony diameter and data are the mean (±SD) of five independent analyses. Columns with different letters at each concentration indicate significant differences according to Duncan’s multiple range tests at p < 0.05.*

### Effects of Thymol/KGM/LG on the Growth Cycle of *Mucor circinelloides*

**FIGURE 4:**
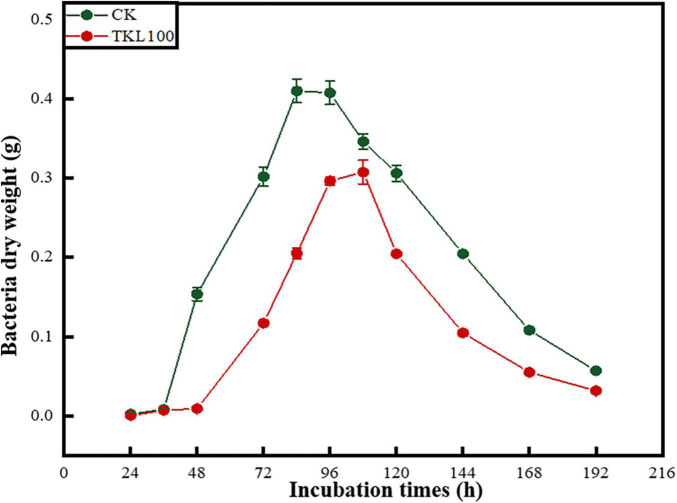
Effects of TKL100 on the growth cycle of *M. circinelloides*.

The growth of *M. circinelloides* could be divided into four stages (Figure 4), which were lagging stage (0–36 h), logarithmic stage (36–84 h), stable stage (84–120 h), and declining stage (120–196 h). *Mucor circinelloides* grew rapidly. When it grew stronger, it would reproduce at about 24 h, entered the growth peak at 36 h, reached the growth limit at 84 h, entered the stable stage, and entered the decline stage at 108 h. However, after the treatment with 100-mg/L TKL edible coating solution, the four growth stages of *M. circinelloides* were delayed hysteresis (0–48 h), logarithmic phase (48–96 h), stationary phase (96–108 h), and declination phase (108–196 h). At 48 h, the growth rate and growth limit decreased, and the dry weight of mycelia was only 75% of that of the control group (*p* < 0.05), the stable period shortens, and the decline rate becomes faster.

### Used Scanning Electron Microscope to Observe the Effect of Thymol/KGM/LG on the Structure of *Mucor circinelloides*

**FIGURE 5:**
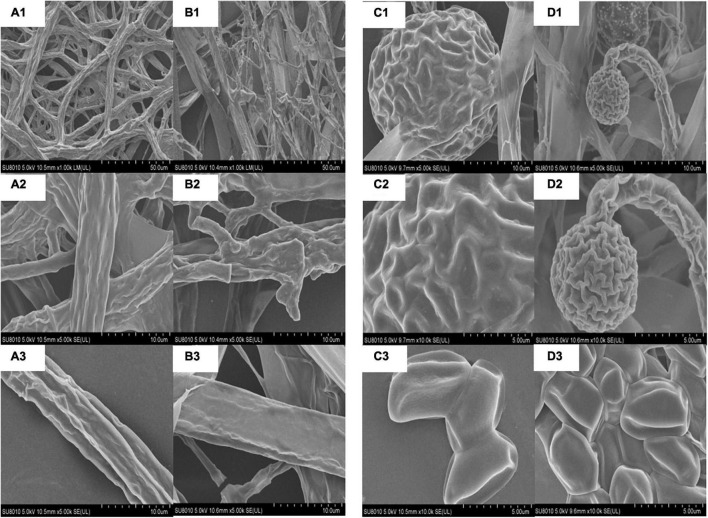
SEM was used to observe the effect of TKL on the structure of *M. circinelloides* **(A–D)**. **(A1–A3)** Healthy mycelia, the magnitudes of 200×, 1,000×, 2,000×; **(B1–B3)** Mycelia treated with TKL100, the magnitudes of 200×, 1,000×, 2,000×; **(C1–C3)** Normal spores, the magnitudes of 200×, 1,000×, 2,000×; **(D1–D3)**, Spores treated with TKL100, the magnitudes of 200×, 1,000×, 2,000×.

A scanning electron microscope (SEM) was used to observe that the normal mycelia of *M. circinelloides* were relatively smooth and club-like, with few curls and folds (Figure 5A). Meanwhile, the spores were smooth and flat as a whole, in the shape of a football (Figure 5C). However, after TKL100 treatment, the mycelia of *M. circinelloides* showed shrinkage, rough and curly surface, and even fracture (Figure 5B). In addition, after the treatment with TKL100, the spores were significantly smaller and their surface was rough and crinkled (Figure 5D).

### Effects of Thymol/KGM/LG on *Mucor circinelloides* Related Enzyme Activities

**FIGURE 6:**
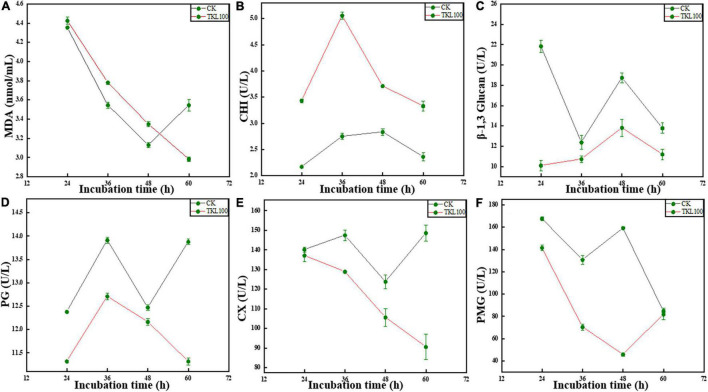
Effects of TKL on *M. circinelloides* related enzyme activities, MDA **(A)**, CHI **(B)**, β-1, 3-glucanase **(C)**, PG **(D)**, Cx **(E)**, and PMG **(F)**. **(A)** After TKL100 treatment for 24, 36, 48, and 60 h, compared with the control group, MDA changes. **(B)** After TKL100 treatment for 24, 36, 48, and 60 h, compared with the control group, CHI changes. **(C)** After TKL100 treatment for 24, 36, 48, and 60 h, compared with the control group, β-1, 3-glucanase changes. **(D)** After TKL100 treatment for 24, 36, 48, and 60 h, compared with the control group, PG changes. **(E)** After TKL100 treatment for 24, 36, 48, and 60 h, compared with the control group, Cx changes. **(F)** After TKL100 treatment for 24, 36, 48, and 60 h, compared with the control group, PMG changes. Error bars are ±SD of the means. In some cases, the error bar is obscured by the symbol. Columns with different letters at each time point indicate significant differences according to Duncan’s multiple range tests at *P* < 0.05.

Under the aging or stress conditions, the microorganisms often undergo membrane lipid peroxidation. Malondialdehyde (MDA) was the final decomposition product of membrane lipid peroxidation, and its content could reflect the degree of stress injury of microorganisms (Wu et al., 2020). See Figure 6A; the MDA content of *M. circinelloides* treated with TKL100 for 12, 24, and 36 h was significantly higher than that of the control group (*p* < 0.05), but significantly lower than the control at 48 h.

The main components of the fungal cell wall are polysaccharides, which mainly included chitin, cellulose, and glucan (Chakraborty et al., 2021). As CHI could catalyze the hydrolysis of chitin, β-1, 3-glucanase could catalyze the hydrolysis of β-1, 3-glucoside bond. Therefore, when the activities of CHI and β-1, 3-glucanase in cells were increased, the important components of the cell wall were reduced and the cell wall structure is damaged (Zhang et al., 2021). In the metabolic process of *M. circinelloides*, chitin hydrolase can also be produced by *M. circinelloides* itself, but the enzyme activity was low and closely related to cell wall degradation. When TKL100 was induced, the activity of chitin hydrolase in cells increased continuously, and with the extension of induction time, the activity of the enzyme increased rapidly at first and then slowly. In the whole treatment stage (see Figure 6B) the CHI activity of the TKL100 group was higher than that of the control group, and the CHI activity of the TKL100 group was about 1.84 times that of the control group, reaching 5.05 U/L (*p* < 0.05). However, after 12, 36, and 60 h treatment with TKL100 (see Figure 6C), β-1, 3-glucanase content was significantly decreased (*p* < 0.05), and there was no significant difference with the control group at 24 h. Therefore, TKL100 may induce the production of CHI to promote the degradation of the *M. circinelloides* cell wall.

Polygalacturonase (PG) and cellulase (Cx) are fungal metabolites that could promote degradation of the plant cell wall, resulting in plant cell damage and disease generation (Pandaranayaka et al., 2019; Osés-Ruiz et al., 2021). As can be seen from the figure, the activity of PG decreased significantly after the treatment with TK100 throughout the whole culture cycle, especially after treatment for 60 h (Figure 6D), the activity of PG decreased by 18.7% compared with the control group (*p* < 0.05). Therefore, TKL100 might inhibit PG synthesis and *M. circinelloides* metabolism. After the treatment with TKL100 (Figure 6E), there was no significant difference in Cx activity at 12 h, but after the treatment with TKL100 for 24, 36, and 48 h, Cx gradually decreased, showing a significant difference from the control group. After 60 h of treatment, the Cx activity of the TKL100 group was only 60.8% of that of the control group, seriously inhibiting the synthesis of Cx. It could be concluded that TKL100 may inhibit the metabolic activities of *M. circinelloides*. Pectin methylgalacturonase (PMG) is also one of the metabolites of mold, which could usually dissolve the cell wall of plant cells, resulting in the rupture and death of the plant cells, resulting in corresponding diseases. After TKL100 treatment (Figure 6F), PMG activity gradually decreased to 28.8% of the control group at 48 h (*p* < 0.05), but at 60 h, the activity of PMG in the TKL100 treatment group began to increase, and there was no significant difference between the TKL100 treatment group and control group.

## Discussion

The isolated pathogens were inoculated into sterilized okra. The results showed that after the inoculation at 28°C for 36 h, brown spots appeared on the okra surface due to pathogen infection. After that, the lesion expanded rapidly, and at 60 h, the surface was wrinkled, softened, depressed, and rotten. The outer skin gradually darkened and turned dark brown. A large number of gray mycelia appeared in the pathogenic site and even spread to the seeds inside okra. Through morphological and molecular identification, the pathogen of okra soft rot was identified as *M. circinelloides*. So far, as far as we know, okra soft rot symptoms and the pathogen *M. circinelloides* have not been reported.

Thymol is extracted from essential oils such as thyme, thyme, oregano, and clove basil. It has antiseptic and antioxidation effects (Nzeako et al., 2006). Studies have shown that thymol has a strong inhibitory effect on a variety of plant pathogens, such as *Magnaporthe Grisea*, *Fusarium graminearum*, etc. (Zhou et al., 2021), followed by thymol has a good antifungal effect on brown rot bacteria (Zhang et al., 2019). Essential oils of thyme and its main components (about 47.59% thymol) (Borugă et al., 2014) have antifungal effects on *aspergillus* and *penicillium* (Sokolić-Mihalak et al., 2012; Pinto et al., 2021). Medina et al. found that chitosan thymol nanoparticles in the edible film had a good inhibitory effect on *Botrytis cinerea* during the storage of blueberry fruits (Medina et al., 2019). Thymol – HP-β-cyclodextrin microcapsules could control acid rot pathogen *Geotrichum Citri-Aurantii* in citrus fruits (Serna-Escolano et al., 2020). The antifungal mechanism of thymol might be the inhibition of pathogen growth and spore germination, the direct effect on phospholipid and protein degradation (Jung et al., 2021), and the inhibition of ergosterol biosynthesis to change the permeability of cell membrane and caused obvious damage to the cell wall and plasma membrane (Galotto et al., 2016), cytoplasmic disintegration and organelle death (Kowalczyk et al., 2020). In addition, some studies have shown that thymol can change cell membrane permeability and inhibit microbial growth by inducing membrane lipid peroxidation (Nagoor Meeran et al., 2017), and it can also inhibit the growth of bocinia cinerea by destroying cell membrane morphology and causing electrolyte leakage (Nazzaro et al., 2013; Folle et al., 2021). In addition, it affects the activity of endometrial proteins such as enzymes and receptors. After entering the cell membrane, thymol interacts with its embedded proteins through various non-specific mechanisms, resulting in changes in conformation and activity of internal and membrane proteins (Khan et al., 2021). Thymol (30 μg/ml) significantly inhibited the mycelial growth and spore germination of *B. cinerea* (Shin et al., 2014).

It was found that TKL could significantly inhibit the growth, reproduction, and spore germination of *M. circinelloides*. At the same time, after 100 mg/L TKL treatment, the *M. circinelloides* delay period was prolonged, 48 h into the growth stage, the growth rate decreased, the growth limit decreased twice, the stable period shortened, the decline rate became faster. A TEM observation showed that compared with the control group, the mycelia of *M. circinelloides* showed the shrinkage with rough surfaces and even fracture after treatment with TKL100, and the spores became significantly smaller with shrinkage and surface depression. These results indicated that TKL could damage the structure of *M. circinelloides* and inhibit spore germination. In addition, the results listed in Table 1 showed that the inhibition rate of the TKL (0) group was –12.01%, which may be because the thymol-free membrane matrix was added to the PDA medium in TKL (0) treatment group. The main raw materials for preparing membrane matrix were polysaccharides, which may provide additional carbon sources for *M. circinelloides*. It promoted the growth and spread of the mycelia of *M. circinelloides*.

To explore the effects of TKL on the *M. circinelloides* membrane, MDA content was determined. The fact that the higher the oxidation level (MDA content) of the mold membrane system, the higher the activity of antioxidative damage-related enzymes was with the accumulation of MDA in cells. Therefore, the MDA content can reflect the injury degree of the mycobacterium somatic membrane to a certain extent. After TKL treatment, MDA content increased significantly at 12–48 h, but decreased sharply at 60 h, even lower than the control, indicating that TKL induced peroxidation of *M. circinelloides* membrane at 12–48 h, resulting in certain damage. However, at 60 h, *M. circinelloides* had a certain stress resistance to TKL stimulation, indicating a potential self-repair mechanism. Therefore, it can be concluded that TKL100 treatment may cause membrane damage of *M. circinelloides* in the early stage of 0–36 h culture, accelerate the degree of peroxidation, and generate more MDA. However, at 48 h, *M. circinelloides* developed resistance to TKL100 and increased tolerance, suggesting a potential self-repair mechanism (Vellanki et al., 2020).

Three enzymes, PG, PMG, and Cx, are the plant cell wall degrading enzymes (CWDE) and can be produced by a variety of plant disease bacteria, usually as metabolites of plant disease bacteria, such as rice sheath blight pathogen *ag1-IA Kuhn* (Datta et al., 2021); the infection of host plants by *Botrytis* spp. Also found that *R. solanacearum* secretes CWDE during host infection, including Cx and PMG (Huang et al., 2019; Yang et al., 2021). The plant disease bacteria metabolize to produce plant cell wall degradation enzymes, resulting in plant cell damage and infiltration, resulting in related plant diseases. After TKL treatment, the activity of PG and Cx of *M. circinelloides* showed a decreasing trend, while PMG showed a decreasing trend first and then increasing. The activity of PG and Cx decreased most significantly at 60 h and decreased most significantly at 48 h PMG. The late decrease of PMG activity may be due to the production of tolerance and stress resistance of bacteria, while the extreme decrease of PMG in the control group may be due to insufficient substrate, and some bacteria begin to decline. Therefore, TKL may inhibit the metabolism of *M. circinelloides*, resulting in the production of its metabolites.

The main components of the fungal cell wall are polysaccharides, including chitin, cellulose, glucan, and so on (Kang et al., 2018; Chakraborty et al., 2021). AS CHI can catalyze the hydrolysis of chitin, β-1, 3-glucanase can catalyze the hydrolysis of β-1, 3-glucoside bond. Therefore, when the activity of CHI and β-1, 3-glucanase is increased, the important components of the cell wall are reduced and the cell wall structure is destroyed. In the metabolic process of *M. circinelloides*, chitin hydrolase can also be produced by *M. circinelloides* itself, but the activity is low and closely related to cell wall degradation. After the TKL treatment, the CHI content of *M. circinelloides* continued to increase, but the β-1, 3-glucanase content did not change regularly after TKL100 treatment. Therefore, TKL100 can also induce the generation of CHI and promote the degradation of the *M. circinelloides* cell wall to achieve the bacteriostatic effect.

## Conclusion

The results showed that *M. circinelloides* was the main pathogenic pathogen of postharvest soft rot of okra. The optimum carbon source of *M. circinelloides* is maltose, and the optimum nitrogen source is beef extract and potassium nitrate. The optimum pH is 6, and it is suitable for the growth of *M. circinelloides* under acidic conditions. The optimum temperature is 15–37°C, and the optimum temperature is 28°C. The low concentrations of NaCl and light were beneficial to its growth. First, the TKL had a good antifungal effect on *M. circinelloides*, and the antifungal mechanism was mainly to inhibit fungal growth, metabolism, and spore germination. Second, it can induce peroxidation of the cell membrane and promote degradation of the cell wall. The TKL is a potential natural bacteriostatic material, which has a good potential in postharvest disease control and storage of okra, but its bacteriostatic mechanism needs further study.

## Data Availability Statement

The raw data supporting the conclusions of this article will be made available by the authors, without undue reservation.

## Ethics Statement

Written informed consent was obtained from the individual(s) for the publication of any potentially identifiable images or data included in this article.

## Author Contributions

QZ and WQ designed the study and revised the final version to be published. QZ, JY, ZW, JD, PH, LL, JW, and XH performed the experiments. QZ drafted the manuscript, wrote, and revised the manuscript. All authors contributed to the article and approved the submitted version.

## Conflict of Interest

The authors declare that the research was conducted in the absence of any commercial or financial relationships that could be construed as a potential conflict of interest.

## Publisher’s Note

All claims expressed in this article are solely those of the authors and do not necessarily represent those of their affiliated organizations, or those of the publisher, the editors and the reviewers. Any product that may be evaluated in this article, or claim that may be made by its manufacturer, is not guaranteed or endorsed by the publisher.
